# Parallel sequencing of *porA* reveals a complex pattern of *Campylobacter* genotypes that differs between broiler and broiler breeder chickens

**DOI:** 10.1038/s41598-019-42207-9

**Published:** 2019-04-17

**Authors:** Frances. M. Colles, Stephen. G. Preston, Kenneth Klingenberg Barfod, Patrik. G. Flammer, Martin C. J. Maiden, Adrian L. Smith

**Affiliations:** 1 Department of Zoology, University of Oxford, The Peter Medawar Building for Pathogen Research, South Parks Road, Oxford, OX1 3SY UK; 2 NIHR Health Protection Research Unit in Gastrointestinal Infections, University of Oxford, Oxford, UK; 3 Present Address: National Research Centre for the Working Environment (NRCWE) (Denmark), Lersø Parkallé 105, 2100 København Ø, Denmark

**Keywords:** Applied microbiology, Microbiome, Bacterial infection

## Abstract

Chicken meat represents an important source of *Campylobacter* infections of humans world-wide. A better understanding of *Campylobacter* epidemiology in commercial chicken flocks will facilitate the development of more effective intervention strategies. We developed a gene-specific parallel sequencing approach that efficiently indicated genetic diversity in farm-derived samples and revealed *Campylobacter* genotypes that would not be detected using microbiological culture. Parallel sequencing of the *porA* nucleotide fragment identified a different pattern of diversity in broiler flocks compared with broiler-breeder flocks at both individual bird and flock levels. Amongst the flocks tested, broiler flocks and individual birds were dominated by one or two *porA* fragment types whereas co-dominance with up to six *porA* fragment types was evident in breeder birds. A high proportion (83.6–93.3%) of *porA* variants were shared between broiler and breeder flocks. The *porA*-based diversity profiling could be a useful addition to the repertoire of tools employed to attribute potential sources of contamination for broiler flocks, including the environment, wild animals or other chickens. This approach can be extended to include other loci within *Campylobacter* and developed for molecular epidemiology studies of other bacterial species.

## Introduction

Campylobacteriosis, predominantly caused by infection with *C*. *jejuni* and *C*. *coli*, is widespread in humans. Attribution modelling suggests that between 58% and 78% of human infection originates from contaminated chicken meat^1,2^. The intestines of broiler chickens become colonized by *Campylobacter* during rearing and this leads to the contamination of carcasses during processing^3^. Reducing the numbers of *Campylobacter* in broiler chickens is critical to reducing the incidence of human campylobacterosis^4^; however, intensive efforts to control this contamination through improved biosecurity at farms and slaughterhouses have been inconsistently effective. A better understanding of the epidemiology of *Campylobacter* transmission into and/or between broiler flocks is key to developing new more effective control strategies. Potential sources of contamination of broiler flocks are widely debated and include other *Campylobacter* positive chicken flocks that are nearby, livestock, wild animals, and water^5–11^.

Typically, *Campylobacter* epidemiology is based upon microbiological culture of the bacterium from different putative sources of contamination, with the most informative studies also employing some form of molecular genotyping of cultured *Campylobacter* (e.g. multi-locus sequence typing or whole genome sequencing)^12–17^. These approaches are constrained by the need to culture and isolate single colonies of *Campylobacter* for molecular analyses and, to date, molecular studies have focused on detailed analysis of a single, or a few colonies from each sample. The ubiquitous presence of *Campylobacter* in poultry environments, the variety of routes by which broiler flocks can become contaminated, the fact that broiler flocks can be colonised by more than one genotype of *Campylobacter*, and the large number of broiler flocks that are reared each year - more than 20 million broiler birds were slaughtered a week in the UK in January to August 2018, means that extensive sampling is required in order to trace routes of infection^14,18–20^. The need for labour-intensive bacteriological culture has limited the depth of analysis in individual birds and flocks. We identified this as a potential problem in defining *Campylobacter* populations and developed a culture independent gene fragment-specific parallel sequencing approach.

A short fragment of the hypervariable *Campylobacter porA* gene, encoding for a major outer membrane protein (MOMP), was chosen as the target for PCR amplification and sequencing using the Illumina MiSeq platform. The nucleotide sequence of the short variable region (SVR) of the *porA* gene has been widely used in typing schemes for *Campylobacter* and found to be highly discriminatory when comparing isolates^21,22^. To determine the *porA* based gene pool of *Campylobacter* in broiler and breeder flocks, the profile of *porA* fragment types were examined in faecal or caecal samples from groups of five individual birds with three replicates for each flock type. The results of the parallel sequencing approach were compared with results obtained by screening up to 50 colony picks, or plate sweeps, from the same samples subjected to microbiological culture.

## Results

### Establishment of a *porAf2* variant identification scheme

The *porA* fragment length used in this study is shorter than that used in previous typing schemes based on Sanger sequencing, and a new scheme to define and catalogue alleles was established on the PubMLST database for *Campylobacter* (https://pubmlst.org/campylobacter/). Data are freely available at this website. Each different *porAf2* variant was given an allele number, based on the order of discovery. The new scheme (designated *porA* fragment 2 or *‘porAf2*’) is compatible with other schemes, meaning that alleles can be assigned from longer nucleotide sequence obtained using the original Sanger sequencing method, or from whole genome sequence^22–24^. The peptide sequence alleles encoded by the *porA* fragment nucleotide sequences were also assigned (designated ‘MOMPf2’) using the *Campylobacter* PubMLST database.

### Campylobacter prevalence

Samples were analysed from four broiler flocks (A, B, C and D, from four different farms,) and three broiler-breeder flocks (E, F and G, from two different farms) that had been identified as *Campylobacter* positive by standard bacterial culture of 20 faecal samples/flock (Table 1). Following a weekly testing schedule, broiler flocks A-C became bacteriologically culture positive at between 32 and 35 days of the rearing cycle whereas *Campylobacter* was detected in broiler flock D only after slaughter at 39 days of age. Breeder flocks E and F both tested positive for *Campylobacter* at 20 weeks when sampling first started, and breeder flock G, sampled from 13 weeks of age, first tested positive at 23 weeks of age.

**Table 1:** Details of the chicken flocks and samples used in the study.

Flock id.	Flock type	Age at sampling (days)		Sample date	
		Faecal samples	Caecal samples	Faecal samples	Caecal samples
A	Broiler	35	37	31.10.2017	2.11.2017
B	Broiler	34	39	25.10.2017	30.10.2017
C	Broiler	32	Not tested	11.10.2017	Not tested
D	Broiler	Not tested	38	Not tested	20.10.2017
E	Breeder	231	Not tested	3.12.2014	Not tested
F	Breeder	420	420	10.4.2017	10.4.2017
G	Breeder	420	424	9.3.2018	13.3.2018

### Parallel sequencing of *porA*

A total of 4,147,590 paired sequence reads were identified as *Campylobacter porA* nucleotide fragments; no *porA* fragments were identified from other bacterial species (Table 2). Between 15,528 and 1,656,017 reads were obtained from faecal and caecal samples for each of the broiler and breeder flocks. Rarefaction analysis indicated that the depth of sequencing within samples captured the diversity of *porAf2* sequences within any individual sample (Supplementary Fig. 1). In total, 1670 different *porAf2* types were identified during the course of this study, of which 1642 variants (98.3%) had not previously been identified on the PubMLST *Campylobacter* database. Due to the presence of indels, the *porAf2* sequences varied in length from 405 to 482 base pairs. The 1670 *porAf2* nucleotide sequences were translated into 1445 different peptide sequences. The ratio of *porAf2* types to number of sequencing reads identified in a sample by parallel sequencing (0.001 to 0.03), was similar to the ratio of *porAf2* types to number of colony picks, identified by microbiological culture (0.008 to 0.02) (Table 2).

**Table 2:** The number and diversity of *Campylobacter porA* fragment genotypes recovered from faecal and caecal contents samples from broiler and broiler breeder flocks using microbiological culture and parallel sequencing.

Flock type	Sample type/ flock id	Microbiological culture		Parallel sequencing, raw data		Parallel sequencing, subsampled data		Simpson’s 1-*D*^b^ (confidence intervals)
		No. *porA* fragment alleles/sequences	Ratio^a^	No. *porA* fragment alleles/sequences	Ratio^a^	No. *porA* fragment alleles/sequences	Ratio^a^	
Broiler flocks	*Faecal samples direct*							
	Flock A	2/171	0.010	60/19,664	0.003	49/10,000	0.005	0.05 (0.04–0.06)
	Flock B	1/193	0.005	112/15,528	0.007	104/10,000	0.010	0.33 (0.31–0.34)
	Flock D	2/231	0.009	43/29,662	0.001	31/10,000	0.003	0.12 (0.12–0.13)
	*Caecal contents samples direct*							
	Flock A	3/146	0.020	183/38,980	0.005	148/10,000	0.010	0.58 (0.57–0.59)
	Flock B	1/127	0.008	140/34,264	0.004	114/10,000	0.010	0.57 (0.56–0.58)
	Flock D	1/217	0.005	33/31,618	0.001	20/10,000	0.002	0.09 (0.09–0.1)
	*Faecal samples plate sweep*							
	Flock A	*N/A*	*N/A*	71/40,665	0.002	50/10,000	0.005	0.04 (0.04–0.05)
	Flock B	*N/A*	*N/A*	108/34,271	0.003	88/10,000	0.009	0.14 (0.14–0.16)
	Flock C	*N/A*	*N/A*	*N/A*	*N/A*	*N/A*	*N/A*	*N/A*
	*Caecal samples plate sweep*							
	Flock A	*N/A*	*N/A*	206/54,416	0.004	202/10,000	0.020	0.99 (0.99–0.99)
	Flock B	*N/A*	*N/A*	133/31,038	0.004	131/10,000	0.010	0.99 (0.99–0.99)
	Flock D	*N/A*	*N/A*	*N/A*	*N/A*	*N/A*	*N/A*	*N/A*
Breeder flocks	*Faecal samples direct*							
	Flock E	*N/A*	*N/A*	785/65,669	0.010	281/10,000	0.030	0.90 (0.90–0.90)
	Flock F	*N/A*	*N/A*	1081/459,011	0.002	240/10,000	0.020	0.85 (0.84–0.86)
	Flock G	2/189	0.010	1198/673,481	0.002	241/10,000	0.020	0.69 (0.68–0.70)
	*Caecal contents samples direct*							
	Flock F	*N/A*	*N/A*	1412/1,656,017	<0.001	322/10,000	0.030	0.83 (0.82–0.83)
	Flock G	2/154	0.010	1251/963,316	0.001	288/10,000	0.030	0.79 (0.78–0.79)

^a^The ratio was calculated by dividing the number of *porA* fragment types by the number of sequences.

^b^A Simpson’s 1-*D* value of 1.0 indicated that each member of a population could be distinguished from every other, and a 1-*D* value of 0 indicated that all members of a population were identical.

### *Campylobacter* diversity in broiler and breeder flocks

Differences in modified Simpson’s Diversity index (based upon normalised total read number) indicated some differences in the composition of *porAf2* variants between the broiler and breeder flocks tested. Faecal samples obtained from broiler flocks had lower indices than breeder flocks (0.05–0.33 compared with 0.69–0.90) and these differences were also apparent with caecal derived samples (0.09–0.58 compared with 0.79–0.83) (Table 2). The same was true when considering the samples from different birds individually (Supplementary Table S1).

**Figure 1:**
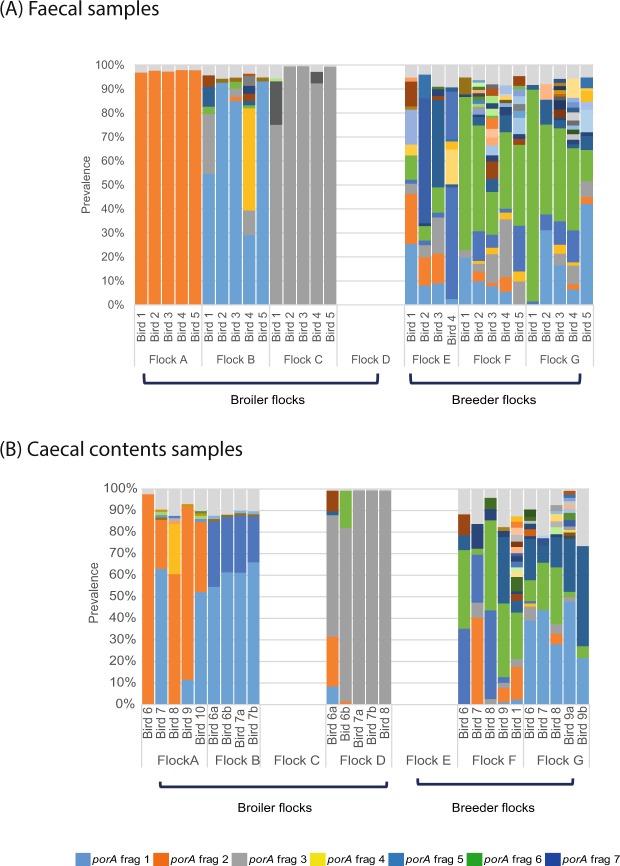
The *Campylobacter porA* genotypes identified by parallel sequencing amongst (**A**) faecal samples and (**B**) caecal contents samples from four broiler flocks and three breeder flocks. Each stacked bar represents the population of *Campylobacter porA* fragment genotypes recovered from samples from individual birds (numbered 1–5 for faecal samples and 6–10 for caecal samples). Each colour represents a different *porA* fragment genotype where present at greater than 1% of the sequences recovered, with dominant *porA* fragment genotypes shown in the key. Where *Campylobacter porA* fragment genotypes had less than 1% prevalence, they were combined and collectively shown in pale grey. Faecal samples were not tested from broiler Flock D, and caecal contents samples were not tested from breeder flocks E and F. Caecal contents samples labelled a and b represent samples collected from the left and right caeca from the same bird.

All except one of the faecal samples tested from three broiler flocks was dominated by a single *Campylobacter porAf2* type, which most often represented more than 90% of all *porAf2* types recovered (Fig. 1A). When caecal contents were tested from birds from the same flocks (but not from the same individual birds as the faecal samples), all except one had a second co-dominant *Campylobacter porAf2* type (Fig. 1B). Caecal contents samples tested from a fourth broiler flock (Flock D, from which faecal samples were not tested) also revealed dominance by one or two *Campylobacter porAf2* types. On five occasions, where left and right caecal samples were analysed from the same bird (annotated as a and b, Fig. 1B) similar, but not always identical, patterns of *porAf2* diversity were evident (Bray-Curtis indices of 0.003–0.31; Supplementary Table S2). Within any broiler flock, almost all faecal or caecal samples from individual birds contained the same dominant *porAf2* type. In contrast, each broiler flock was generally dominated by a different *porAf2* type.

Faecal samples tested from three breeder flocks had between two and six co-dominant *Campylobacter porAf2* types per individual bird. Similarly, caecal contents samples tested from two breeder flocks had between two and five co-dominant *porAf2* types per individual bird (Fig. 1A,B). Only one of 24 faecal and caecal contents samples tested from breeder birds showed the pattern of dominance by a single *Campylobacter porAf2* type. In addition to the increased number of co-dominant *porAf2* types within individual birds the pattern of co-dominant *porAf2* types differed between birds within the same flock. This was supported by both the between-bird-within-flock, and the between-flock Bray Curtis dissimilarity figures being much higher for breeder flocks (0.25–0.67) than broiler flocks (<0.001 to 0.77 with 42/52, 80.8% of values below 0.39) (Supplementary Tables S2 and S3). A Mann-Whitney U test comparing *porAf2* genotype populations between the broiler and breeder flocks gave a value of U = 3.27, *p* < 0.0001, rejecting the null hypothesis that the broiler and breeder flocks have the same *Campylobacter* gene pool.

### Parallel sequencing identified micro-variants of *Campylobacter porAf2 types*

**Figure 2:**
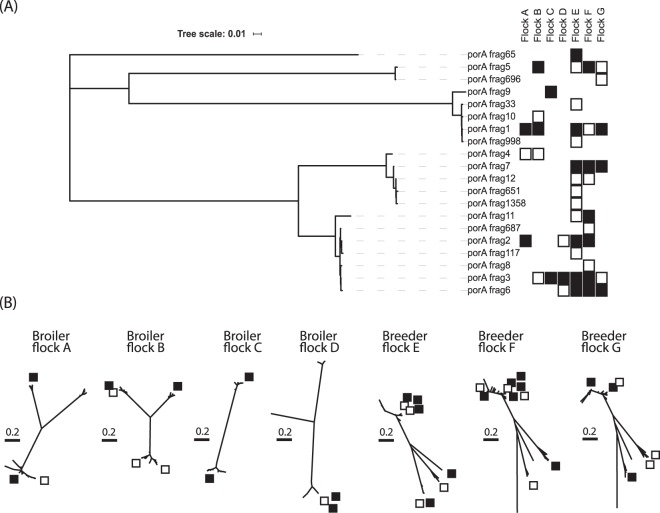
Neighbour joining trees showing the evolutionary distances between (**A**) the dominant *Campylobacter porA* fragment genotypes amongst each of the broiler flocks (A to D) and breeder flocks (E–G) and (**B**) all of the *Campylobacter* fragment *porA* genotypes identified amongst each of the flocks individually. The *porA* fragment genotypes with greater than 5% prevalence are marked with a filled black box, and the *porA* fragment genotypes with greater than 1% prevalence are marked with a white box.

Although birds in broiler flocks were typically dominated by one or two *porAf2* type(s) and breeder flocks by up to six co-dominant types (at >5% of total) a large number of low frequency, variant *porAf2* types were detected by parallel sequencing. The analysis pipeline retained only sequences found on 10 or more occasions across the dataset to minimise the effect of sequencing errors, and within any flock these rare variants of the *porAf2* were often found in more than one sample, i.e. derived from more than one bird. The numbers of low frequency variants detected within flocks ranged between 20 and 148 for broiler flocks and 240 and 322 for breeder flocks (Table 2). Neighbour joining trees (Fig. 2) demonstrated that for each of the samples, the dominant and co-dominant genotypes formed a number of distinct genetic lineages, whilst the majority of the rare variants were very closely related to the dominant and co-dominant *Campylobacter porAf2* types, most often representing a single nucleotide polymorphism. The nucleotide changes were detected across the length of the fragment, but they did not occur at every base and the majority (1445/1674, 86.3%) resulted in a new peptide sequence.

Rarefaction curves (Supplementary Fig. S1) demonstrated that sufficient sequencing depth was achieved in the study to recover most of the diversity of the *Campylobacter porAf2* types present in each of the samples tested from individual birds. At a flock level the rarefaction curves for broiler faecal or caecal samples plateaued at between 50 and 100 *porAf2* variants whereas for breeder flocks the rarefaction curves were only beginning to plateau at around 1000 to 1500 variants. The cumulative rarefaction curves using the 5 individual birds/flock suggested that this level of sampling captured the majority of genotypes present in broiler flocks (the lines overlie each other). In contrast, for the breeder flock faecal samples the stepped pattern indicated that adding more birds increased diversity of the detected *porAf2* types. This was less evident with caecal samples.

### Comparison between parallel sequencing and microbiological culture

#### Multiple colony picks

**Figure 3:**
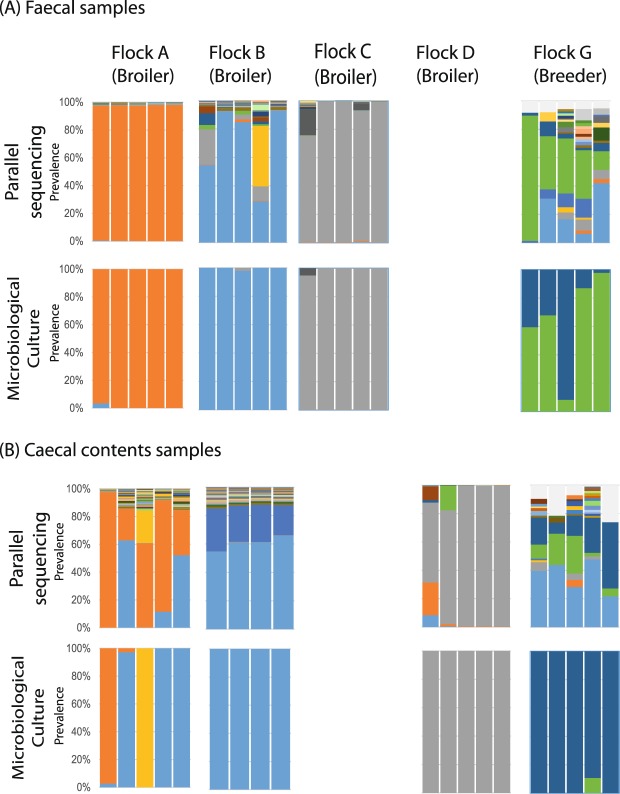
Comparison of the *Campylobacter porA* fragment genotypes identified amongst (**A**) faecal and (**B**) caecal contents samples from four broiler flocks and one breeder flock by parallel sequencing (top panels) and matched multiple colony picks from microbiological culture (bottom panels). Each bar represents the population of *Campylobacter porA* genotypes recovered from a sample collected from an individual bird, and each colour represents a different *porA* genotype. For both faecal and caecal contents samples, a bar in the top panel showing the *Campylobacter porA* fragment genotypes recovered from a sample by parallel sequencing is directly matched to the bar immediately below showing the *Campylobacter porA* fragment genotypes recovered from the same sample using microbiological culture. Faecal samples were not tested from flocks D, E and F, and caecal contents samples were not tested from flocks C, E and F.

The dominant *Campylobacter porAf2* type identified using the parallel sequencing approach was recovered for each of the broiler flock faecal samples tested using multiple colony picks from microbiological culture (Fig. 3). In all except three of the 15 faecal samples tested from broiler flocks, the dominant type was the only one recovered. In three faecal samples, a second *porAf2* type was recovered at low frequency. For example, faecal samples from birds 1 and 4 in broiler flock B, revealed more than one dominant *porAf2* type by direct parallel sequencing, whereas the colony pick approach only identified a single type. For faecal samples from breeder flock G the circumstance was more complex. Two of the dominant *porAf2* types were detected using both methods but the colony pick approach underestimated the overall diversity in these birds compared to direct sequencing. In addition, the proportions of the colony pick detected genotypes did not match those detected by parallel sequencing.

The results from the broiler caecal contents samples, most often containing two co-dominant *porAf2* types by parallel sequencing, were less consistently in agreement with colony pick results than for the broiler faecal samples. Whilst at least one of the co-dominant *Campylobacter porAf2* types was recovered from the multiple colony picks from caecal contents samples, all but two of the colony pick analyses identified a single *porAf2* type. For example, within caecal samples from broiler flock A the *porAf2-2*, type which was co-dominant in all five birds, was only detected in culture from bird 1 where it was the only genotype, and as a tiny proportion (<5%) in bird 2. For bird 3 in flock A, *porAf2-*4 type which represented ~20% of the parallel sequencing genotypes was the only genotype detected by the colony pick approach. Within caecal samples from broiler flock B, *porAf2-5* type was not detected by colony pick despite being present in all four birds by parallel sequencing. With caecal samples from broiler flock D, three of the birds had a single dominant *porAf2* type by either approach; however, other constituent genotypes detected at between 5–15% by parallel sequencing were not detected by colony pick. For caecal samples from breeder flock G, which contained diverse co-dominant genotypes by parallel sequencing, results from the colony pick approach were dominated by the same single genotype in all five birds. This genotype was one of the co-dominant genotypes, varying between ~10–40% of *porAf2* genotypes identified by parallel sequencing, yet other co-dominant strains were not identified by colony pick genotyping.

#### Plate sweep approach

**Figure 4:**
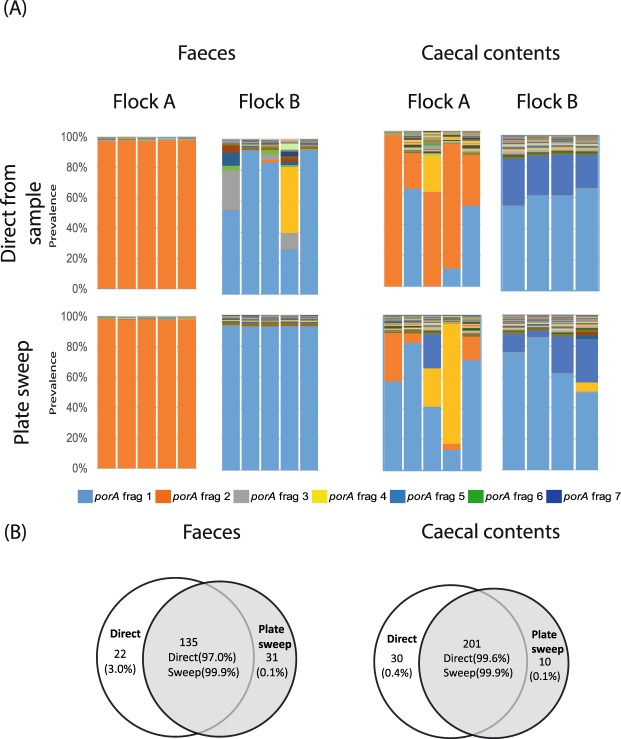
*Campylobacter porA* fragment genotypes identified by parallel sequencing compared between faecal and caecal contents (top panels) and sweeps of bacterial growth (‘plate sweep’) from the same samples on *Campylobacter* selective agar (bottom panels) for broiler flocks A and B. All *Campylobacter porA* fragment genotypes are shown for each sample type, with each stacked bar representing the population identified from an individual bird. For each set of panels, the plate sweep results for a given sample are shown directly beneath the direct sequencing results. Venn diagrams are depicted (**B**) displaying the proportion of *porA* genotypes detected by direct sequencing of faecal or caecal samples compared with those detected by parallel sequencing of the cultured “plate sweep” samples. The % of total reads accounted for by the sequences that fall within each segment is given in brackets for both direct and plate sweep samples.

The plate-sweep approach, where *porA* parallel sequencing was performed using the DNA from all bacterial growth from mCCDA plates for samples from broiler flocks A and B, identified a broader range of *Campylobater porAf2* types of between 45 and 85 genotypes/faecal sample, and 61 and 454 genotypes/caecal sample, which matched the direct sequenced samples with more accuracy than the colony pick approach (Fig. 4). These included rare genotypes that comprised <1% of the genotypes detected by direct sequencing of the faecal samples from both flock A and flock B. The plate sweep approach also produced a closer representation of the direct sequence diversity with the caecal samples, although with some samples there was evidence that some *Campylobacter porAf2* genotypes grew more efficiently on agar than others (e.g. the *porAf2-4* type in birds 3 and 4 from broiler flock A). The number of distinct *porAf2* genotypes detected using direct sequencing or plate sweep-sequencing of individual birds were similar and contained highly overlapping populations of genotypes (97.0–99.9% of sequences) for both sample types from flock A and flock B (Fig. 4). The Bray-Curtis dissimilarity index values obtained when comparing direct *porAf2* sequencing from samples with sweep sequencing were below 0.29 with the exception of three birds, which gave values of 0.50 to 0.68 (Supplementary Table S4). A Mann-Whitney U test comparing the *porAf2* diversity between the direct samples and plate sweeps gave a U value of 28725, *p* 0.20, consistent with the null hypothesis that they shared the same *Campylobacter porAf2* variants.

### Patterns of shared and distinct genotypes revealed by direct *porA* parallel sequencing

**Figure 5:**
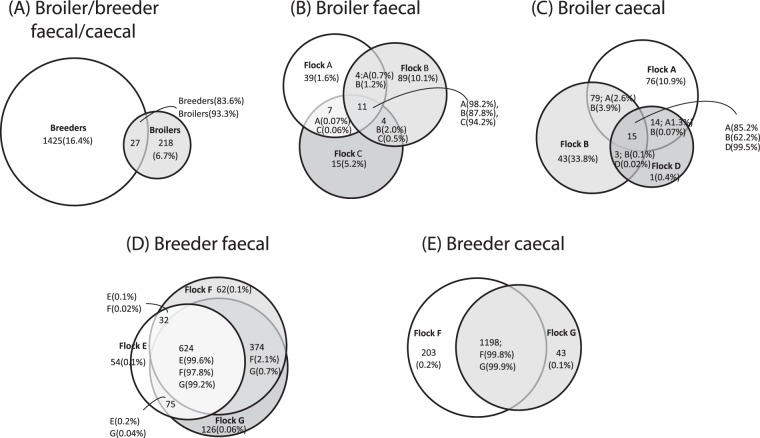
Venn diagrams showing the overlap between *Campylobacter porA* fragment genotypes isolated from the different sources; (**A**) all faecal and caecal contents samples from broiler and breeder flocks, (**B**) faecal samples from broiler flocks A-C, (**C**) caecal contents samples from broiler flocks A, B and D, (**D**) faecal samples from breeder flocks E,F and (**E**) caecal contents samples from breeder flocks F and G. The number of shared *porA* types are indicated within segments and the percentage of total sequence reads that each subset of *porA* types represent are given in brackets.

During the course of this study, we identified 1670 different *porAf2* types in four broiler flocks and three breeder flocks with 27 genotypes found in both types of flock (Fig. 5). The 27 genotypes comprised 83.6% of the total number of *porAf2* types detected in breeder flocks, and 93.3% of those seen in broiler flocks (Fig. 5A). Similarly, when considering faecal samples from three broiler flocks from different farms (A, B and C), a small number (11/169) of *porAf2* types were detected in all three flocks and all of the genotypes that were dominant in each individual flock were detected in all three flocks. Indeed, the 11 common *porAf2* types represented between 87.8% and 98.2% of the sequences seen in individual flocks. A further 15 sequences were detected in samples from more than one farm. A very similar pattern was evident with the caecal samples from 3 broiler farms (A, B and D) with 15/231 types found in all three farms and these 15 represent the majority of total *porAf2* types detected.

A similar analysis for the three breeder flocks revealed a different pattern. In this case flocks E and F were from the same farm separated by time (in 2014 and 2017) and flock G was from a different farm (2018). A large number of genotypes (624 of 1347) were present in all three flocks and these represented between 97.8 and 99.6% of all *porAf2* genotypes detected in each of the three farms, with a further 374 shared between flock F and flock G. Breeder-derived caecal samples were only available for flocks F and G with 1198/1444 detected types being shared between both flocks (located at different farms) representing almost all (99.8 and 99.9%) of the sequences detected in each of the farms.

## Discussion

*Campylobacter* causes one of the most common foodborne zoonoses with transmission to humans most often linked to consumption of contaminated poultry products^1,25^. A 2008 survey found 71% of EU broiler chicken flocks to be *Campylobacter* positive at slaughter and despite concerted efforts, a reduction in the incidence of *Campylobacter* has proven very difficult to attain^26^. As obligate microaerobic bacteria, their persistence and ability to survive from farm to retail outlets is puzzling^27^. Labour intensive, culture-based molecular analyses typically focus on single, or a few colonies, per sample and therefore may not fully evaluate genetic diversity within samples, flocks, and individual birds. We applied a new approach, which focussed on a culture-independent assessment of *Campylobacter* genetic variation by direct amplification of a fragment of the hypervariable *porA* gene, followed by parallel sequencing.

Parallel-sequencing of the *porA* fragment amplicon identified 1670 *porAf2* sequences amongst the samples tested in this study, many of which represented new *porAf2* types. The small number of dominant *porAf2* types identified amongst the flocks tested had been previously identified on the PubMLST *Campylobacter* database and may represent those variants that most successfully colonise and transmit among poultry flocks^28^. The majority of low frequency *porAf2* types were closely related to the dominant genotypes at the nucleotide level, but often encoded a new peptide sequence. The PorA protein is thought to be under immune selection, and at least some of the low frequency variants may represent antigenically distinct variants^21^. Longitudinal sampling within individual birds, not easily possible under field circumstances, may identify fluctuations in the frequency of *porA* variants that relate to immune-mediated selection over time. Experimental infections with mixtures of *Campylobacter* strains did report a switch in the dominant strain at 27–30 days of age, which would be consistent with changes in population structure associated with immune pressure^29^. The *porAf2* type was used as an indicator for *Campylobacter* genotype in this study. Whilst *Campylobacter* is genetically diverse, population studies based on multilocus sequence typing of housekeeping genes have identified more than 43 clonal complexes, many of which are associated with particular isolation sources^21^. Non-random association between *porA* fragment variants and clonal complexes have been demonstrated for *Campylobacter*, although the associations are not exclusive or predictive. *Campylobacter* strains with different *porA* fragment types are generally distinct at other loci, but it should be noted, that those with the same *porA* fragment type may also represent different strains, that would be apparent if additional genes were to be tested.

The parallel sequencing method also indicated differences in the *Campylobacter porAf2* variants from the broiler and breeder flocks tested in this study. The broiler flocks were dominated by one or two *porAf2* types, whereas the breeder flocks contained a much greater diversity. Within each broiler flock, tested just before slaughter, the one or two dominant genotypes comprised >80% (and often >95%) of the detected sequences, with a number of additional rare variants in each bird. Different birds within any broiler flock were typically very similar, dominated by the same *porAf2* genotype as other members of the flock, as was identified in culture based studies that examined a single colony per sample^14,30^. Notably, the dominant *porAf2* genotype usually differed between the broiler flocks, although the dominant genotypes in one broiler flock were identified as a rare component of other flocks. Cumulative rarefaction analysis indicated that there was little to be gained by sampling more than five birds from a broiler flock, which typically represents upwards of 20,000 individuals. The “simple” pattern of *Campylobacter porAf2* diversity in broiler flocks may suggest that contamination of the flock is a rare event, with the successful strain expanding in the flock during the growth period.

Breeder flocks and the individual birds within these flocks contained a more diverse *porAf2* gene pool than broilers, with evidence of co-dominance of up to 6 genotypes in each bird. The bird-to-bird variation was also much higher in the breeders than broilers. The breeder birds were much older than the broiler birds and the *Campylobacter* diversity is likely to be at least in part due to age^31,32^. Feed represents another important difference between breeders and broilers and this is known to affect the numbers of *Campylobacter* in single-strain initiated experimental systems^32^. In contrast to broiler flocks, the cumulative rarefaction analysis indicated that sampling additional birds increased detectable diversity in breeder birds.

Caecal samples from broiler birds consistently contained more variation in *porAf2* genotypes (up to three types >10%) compared with faecal samples. Notably, the same *porAf2* genotype was identified as most frequent in both faecal and caecal samples taken from any individual broiler flock. Our findings are consistent with those from a previous study that identified matching MLST types in faecal and caecal samples from broilers^14^. The additional variation in caecal versus faecal diversity detected in our study may reflect the fact that not all parts of the gut contribute to any individual faecal sample. For instance, some faecal samples are derived from a greater proportion of caecal material than others, indeed the caeca are blind-ended muscular pouches that periodically empty into the main digestive tract and it is possible to differentiate caecal-derived from non-caecal derived faecal material by colour and consistency. Microbiologically, the caeca can be sites where greater numbers of bacteria, including *Campylobacter*, are located^33,34^. The importance of sampling different types of material should be taken into account, depending upon the question being considered. In many cases non-invasive longitudinal sampling of faeces could be considered appropriate and these contain the most common *Campylobacter* genotypes.

When comparing the parallel sequencing results with microbiological culture, the dominant *porAf2* type(s) were detected by both methods in all of the samples tested. There was some evidence of competitive over-representation of some *porAf2* types following culture, and the proportional distribution of different *porAf2* types was not always consistent between the two methods. This was particularly evident amongst broiler caecal samples, and faecal and caecal samples from breeder flocks, where the colony pick approach did not represent all of the co-dominant *porAf2* types identified by parallel sequencing. The *porAf2* profiles obtained from plate-sweeps (DNA sequenced from all bacterial growth on a plate) provided a much closer match to the direct sequencing profiles than colony picking, indicating that most *porAf2* sequences were derived from culturable *Campylobacter*. These results highlight the potential for variation among studies that use different culture media and criteria for isolating *Campylobacter* for downstream analyses^35–38^.

Identifying the source of *Campylobacter* contamination in poultry of all types remains complex but very important in terms of developing new, more effective strategies to reduce poultry-related risks to human health^6,8,39^. We have shown the *porA* parallel sequencing approach to be an efficient and sensitive means by which the genetic diversity of *Campylobacter* can be investigated within samples from different types of chicken flock. This approach offers a new perspective that will impact our understanding of *Campylobacter* epidemiology, fitting alongside the current molecular-based MLST and whole-genome sequencing methods. It can be developed to target other loci within *Campylobacter* (eg. *flaA)*, and may also be applicable to studying the molecular epidemiology of other human and animal pathogens.

## Materials and Methods

### Samples

Twenty fresh faecal samples were collected from live birds on the farm for each of three broiler flocks (flocks A, B and C) and two broiler breeder flocks (flocks F and G), shortly before they were taken for slaughter (Table 1). Similarly, twenty fresh faecal samples were collected from live birds from a third broiler breeder flock (flock E) aged 33 weeks, mid-way through their production cycle. From the sets of twenty faecal samples, five were chosen for each flock where separate *Campylobacter* colonies could sub-cultured. The broiler flocks were all sampled within 20 days of each other in the month of October 2017 (Table 1). The broiler breeder flocks were sampled in different years between 2014 and 2017, two in spring and one in winter. All flocks were housed and from the same company. All of the flocks were from different farms, with the exception of broiler breeder flocks E and F which were separated by another broiler breeder flock (not tested) in between. The broiler flocks were a standard commercial breed, mixed sex and thinned before slaughter.

Each of the faecal samples were collected from different areas across the house, to maximise the diversity of *Campylobacter* genotypes recovered, and were transported to the laboratory by post or courier at room temperature. Caecal samples were collected from three of the broiler flocks (A, B and D) at 37 to 39 days of age and also from two of the breeder flocks (F and G) aged 420–424 days and transported chilled to the laboratory. Five birds were chosen during the middle of processing of the flock to reduce the possibility of cross contamination from other flocks. It is unlikely they were the same individuals from which faecal samples were collected, but they were from the same house on the farm. It was not possible to collect caecal samples from flocks C and E, and faecal samples from broiler farm D were culture negative for *Campylobacter* before slaughter. All of the faecal samples and caecal contents were processed in the laboratory within 48 hours of collection, and then immediately stored at −80 °C prior to DNA extraction.

### Microbiological culture

The faecal and caecal contents samples were stirred immediately before inoculating the tip of a swab onto mCCDA and spreading for single colonies. Both left and right caecal for a bird were cultured individually. The inoculated agar plates were incubated at 42 °C for 48 hours in a microaerobic atmosphere using the GenBox Microaer system (BioMerieux Ltd, UK). Presumptive *Campylobacter* colonies were identified by characteristic appearance, Gram, catalase and oxidase reactions. Where colonies formed distinct, rather than spreading colonies, up to five samples per flock were selected for subculture of between 22 and 50 colonies onto separate Columbia Blood agar plates (Oxoid, UK). These plates were then incubated for a further 48 hours at 42 °C in a microaerobic environment and examined for purity. The identity of each of the colonies was confirmed to be *Campylobacter* using *porA* short variable region (SVR) Sanger sequencing^22^. In addition, all bacterial growth from the mCCDA plate, referred to as a ‘plate sweep’, was harvested in 400 µl TE buffer for each of the samples and immediately stored at −80 °C.

### Sanger sequencing

Chromosomal DNA was extracted from the pure *Campylobacter* cultures by preparing dense cell suspensions in 250 µl PBS. These were placed in a boiling water bath for 10 minutes and then spun in a micro-centrifuge at 13,200 rpm for 5 minutes. The supernatant was retained and cellular debris discarded. Sanger sequencing was performed as previously published^13,22^. Briefly, 25 µl PCR reactions were performed in 96 well microtitre plates using the MOMP 3 F and 2 R primers^22^. PCR reactions were precipitated using PEG-NaCl and washed twice using cold 70% ethanol. 10 µl sequencing reactions were performed using BigDye V3 and the MOMP 3 F and 2 R primers. Sequencing reactions were precipitated using Sodium Acetate pH 5.2, and washed once using cold 70% ethanol. The sequencing reaction products were separated on an ABI 3730 automated DNA analyser. The DNA sequences were assembled and trimmed using the STARS and Staden software packages (ref). The *porA* fragment alleles were assigned using the PubMLST *Campylobacter* database (https://pubmlst.org/campylobacter/).

### Parallel sequencing

DNA was extracted directly from frozen faecal and caecal contents samples using the Maxwell 16 automated DNA extraction system (Promega, UK), alongside extraction controls containing lysis buffer only. The extractions were performed in a separate room from which *Campylobacter* was cultured and equipment was cleaned using DNA Zap (SigmaAldrich, UK) and 70% ethanol prior to use. From the frozen samples, 250 mg was scraped into individual microcentrifuge tubes containing a quarter volume of 0.5 mm zirconia/silica beads (Thistle Scientific, UK) and 600 µl lysis buffer from the Maxwell 16 LEV Blood kit (AS 1290, Promega, UK). The samples were put in a bead beater at high speed for 1 minute, and then heated at 95 °C for 10 minutes. They were then centrifuged at 13,200 rpm in a micro-centrifuge for 5 minutes before treatment with proteinase K at 56 °C for 20 minutes. The supernatant was then loaded into the Maxwell 16 LEV Blood kit cartridge on the Maxwell 16 DNA extraction robot, and DNA extraction performed following the manufacturer’s ‘Blood’ LEV extraction protocol.

DNA was extracted from ‘plate sweep’ samples using the Maxwell 16 Cell DNA purification kit (AS 1020, Promega, UK), alongside DNA extraction controls containing TE buffer only. All of the frozen plate sweep cell suspension in 400 µl TE buffer for each sample was thawed on ice, and then loaded into the Maxwell 16 Cell DNA purification kit cartridge on the Maxwell 16 DNA extraction robot. DNA extraction was performed following the manufacturer’s ‘SEV Blood/Cells’ protocol.

Presence of DNA in each of the extracted samples was confirmed using the Qubit. It was necessary to dilute the DNA 1:10 before use to overcome the effect of PCR inhibitors.

In a third ‘clean’ room, specifically purposed for PCR, 25 µl reactions were set-up using a newly designed MOMP B primer 5′-CCA CAA TTA TGG TTA GCT TA-3′ and one per reaction of 100 different MOMP 2 R primers tagged with 7 nucleotide ‘barcode’ sequences, allowing multiplexed reactions within sequencing libraries to be identified during downstream bioinformatics processing^22^. More than 2,000 *porA* short variable region sequences held on the http://pubMLST.org/campylobacter database were screened in order to design the MOMP B primer to amplify all variants. The high fidelity Phusion Hot Start Flex DNA polymerase enzyme (New England Biolabs, UK, M0535) was used according to the manufacturer’s recommendations. An annealing temperature of 58 °C was found to be optimal, giving the following thermocycling conditions; initial denaturation 98 °C for 30 seconds, 35 cycles of 98 °C for 10 seconds, 58 °C for 30 seconds, 72 °C for 30 seconds, and final extension of 72 °C for 10 minutes.

Up to 100 PCR reactions from different samples with individually tagged MOMB 2 R primers were combined with indexed primers to make a library to run on the MiSeq machine in a fourth separate room. From each of the triplicate PCR reactions, 2 µl was combined, and then cleaned using the MinElute PCR purification kit (28004, Qiagen, UK). Library preparation was performed using the NEB ultra DNA Library Prep kit for Illumina (E7370, New England Biolabs Inc, UK), following the manufacturer’s instructions, with no size selection. Indexing primers (New England Biolabs Inc, UK E7335S and E7500S) were used in order to run several different libraries on the MiSeq machine in parallel. TapeStation (Agilent Genomics) and qPCR (E7630L, New England Biolabs, UK) analyses were used to confirm expected template size and success of the library preparation before loading onto the MiSeq along with 5% phiX. The 600-cycle MiSeq Reagent Kit v3 (Illumina, UK, MS-102-3003) was used, giving paired 300 nucleotide reads.

### Downstream processing of MiSeq data

Barcoded reads were demultiplexed using custom Python scripts, and terminal primer sequences trimmed. Any reads still containing a copy of either primer sequence after this trimming were identified using cutadapt v1.15, and excluded from further analyses on the assumption that they represent PCR artefacts (e.g. concatamers)^40^. The fastq_eestats2 command in USEARCH v10.0.240 as used to show that 3′-trimming to 270 bp to would adequately remove low quality sequence^41^. DADA2 1.8 was then used to trim reads, filter to <=1 expected errors/read, assign sequence variants (using pooled sequences), merge read-pairs, and remove chimeras (using the removeBimeraDenovo() function with method = “consensus”)^42^.

The *porA* SVR fragment nucleotide sequences were aligned in MEGA to confirm they were legitimate coding sequence for the correct species and gene fragment – all were from the *Campylobacter porA* SVR as expected^43^. The first nucleotide was trimmed so that the sequence was in open reading frame to give an equivalent amino acid sequence. The 3′ end of the sequence was trimmed by approximately 20 nucleotides to match *porA* SVR alleles that were previously defined on the PubMLST *Campylobacter* database (https://pubmlst.org/campylobacter/), for a Sanger sequencing based method, making the two systems compatible^23^. A small number of sequences containing stop codons were removed from analysis. The nucleotide sequences were also translated into amino acid sequence using MEGA. Each of the *porAf2* alleles and corresponding PorAf2 peptide sequences were added to a new scheme on the PubMLST database making them publically available. Since it is impossible to tell apart rarely occurring sequencing errors, and rarely occurring *porAf2* sequences, a conservative approach was taken, removing all *porAf2 *sequences that occurred 10 times or fewer across the total data set from further analysis.

### Diversity and dissimilarity indexes

A modified Simpson’s diversity index was calculated for each of the sample types (faecal, caecal and plate sweep samples) where available, for each of the 4 broiler farms and 3 breeder farms^44^. They were calculated both for raw data and for data that had been subsampled using the stochastic method employed by USearch v11^41^. A 1-*D-*value of 1.0 indicated that each member of a population could be distinguished from every other, and a 1-*D-*value of 0 indicated that all members of a population were identical. Calculations were performed using the Paleontological statistics (PAST) software package^45^.

The Bray-Curtis dissimilarity index was used to compare the populations of *Campylobacter porAf2* genotypes between birds from the same flock (alpha diversity) and between birds from the different flocks (beta diversity) using data subsampled to the lowest denominator of sequencing reads recovered. For alpha diversity calculations of individual birds, reads were subsampled to 1,000 for faecal, caecal and plate sweep samples from broiler birds, and to 19,000 for faecal samples and 45,000 for caecal samples from breeder birds. For beta diversity calculations, all sets of samples were subsampled to 10,000 reads. A Bray-Curtis value of 0 meant the two  flocks have the same *Campylobacter porAf2* distribution, and a value of 1 meant the two flocks did not share any *porAf2* alleles^46^. The Bray-Curtis calculations were performed using the R software ‘vegan’ package^47^.

### Other analyses

Analyses other than the diversity and dissimilarity indexes were performed using raw, rather than subsampled data. Rarefaction curves were generated using PAST software for (a) samples combined for each farm and sample type, and for (b) to show cumulative effect of adding results from consecutively sampled birds on a farm for each sample type^45^. A non-parametric, two tailed Mann-Whitney test to compare the distribution of *porAf2* populations between broiler/breeder flocks, and direct/plate sweep samples was also performed using PAST software.

Neighbour joining trees were generated using MEGA 7 software and the maximum composite likelihood method^43^ and displayed using Figtree v1.4.3 (http://tree.bio.ed.ac.uk/software/figtree/). The neighbour joining tree in Fig. 2, panel (A) was annotated using the Interactive Tree of Life (iTOL) 4.2.3 software package^48^.

### Ethical approval

This study used broiler flocks that were reared commercially by industry partners, in line with standard industry practice. We performed non-invasive microbiological sampling and thus the need for approval under the Animals (Scientific Procedures) Act of 1986 was waived. All prevailing local, national and international regulations and conventions, and normal scientific ethical practices have been respected.

## Supplementary information

Supplementary Dataset 1
