# Supplementary material for: Parallel sequencing of porA reveals a complex pattern of Campylobacter genotypes that differs between broiler and broiler breeder chickens
Source: Sci Rep. 2019 Apr 17;9:6204. doi: 10.1038/s41598-019-42207-9 (PMC6470227; doi:10.1038/s41598-019-42207-9)

Supplementary data

Parallel sequencing of *porA* reveals a complex pattern of *Campylobacter* genotypes that differs between broiler and broiler breeder chickens.

Frances M. Colles[1*], Stephen G. Preston[1], Kenneth Klingenberg Barfod[1a,] Patrik G. Flammer[1], Martin C. J. Maiden[1,2] , Adrian L. Smith[1]

Table S1. The diversity of *Campylobacter porA* fragment genotypes identified amongst individual faecal and caecal contents samples from broiler and breeder birds. A Simpson's Diversity 1-*D* value of 1.0 indicated that each member of a population could be distinguished from every other, and a 1-*D* value of 0 indicated that all members of a population were identical. There were no faecal samples tested from flock D or caecal samples tested from flocks C and E and '-' indicates no sample. Paired caecal samples from the same bird were tested from five birds across flocks B, D and G, with 'a' and 'b' indicating left and right caeca.

**Faecal Samples**							
	**Flock A**	**Flock B**	**Flock C**	**Flock D**	**Flock E**	**Flock F**	**Flock G**
Bird 1	0.06 (0.05-0.08)	0.63 (0.60-0.65)	0.40 (0.39-0.42)	-	0.85 (0.85-0.85)	0.55 (0.55-0.55)	0.22 (0.22-0.22)
Bird 2	0.05 (0.01-0.05)	0.14 (0.13-0.15)	0.01 (0.01-0.02)	-	0.69 (0.69-0.70)	0.77 (0.77-0.78)	0.74 (0.74-0.74)
Bird 3	0.05 (0.05-0.06)	0.28 (0.24-0.31)	0.01 (0.01-0.01)	-	0.81 (0.80-0.81)	0.93 (0.93-0.93)	0.80 (0.80-0.80)
Bird 4	0.04(0.02-0.07)	0.72 (0.70-0.74)	0.15 (0.13-0.16)	-	0.72 (0.72-0.73)	0.82 (0.82-0.83)	0.84 (0.84-0.84)
Bird 5	0.04 (0.04-0.05)	0.13 (0.12-0.15)	0.02 (0.01-0.02)	-	-	0.83 (0.83-0.83)	0.78 (0.78-0.79)
**Caecal contents samples**							
	**Flock A**	**Flock B**	**Flock C**	**Flock D**	**Flock E**	**Flock F**	**Flock G**
Bir d 6	0.05 (0.04-0.06)	-	-	-	-	0.72 (0.72-0.73)	0.77 (0.79-0.79)
Bird 7	0.55 (0.54-0.56)	-	-	-	-	0.80 (0.80-0.80)	0.75 (0.75-0.75)
Bird 8	0.58 (0.57-0.59)	-	-	0.01 (0.01-0.02)	-	0.65 (0.65-0.65)	0.82 (0.82-0.82)
Bird 9	0.35 (0.33-0.36)	-	-	-	-	0.78 (0.78-0.78)	-
Bird 10	0.62 (0.61-0.63)	-	-	-	-	0.91 (0.91-0.91)	-
Bird 6a	-	0.61 (0.60-0.62)	-	0.61 (0.59-0.64)	-	-	-
Bird 6b	-	0.56 (0.55-0.57)	-	0.64 (0.32-0.31)	-	-	-
Bird 7a	-	0.56 (0.54-0.57)	-	0.01 (0.01-0.02)	-	-	-
Bird 7b	-	0.52 (0.51-0.53)	-	0.01 (0.09-0.02)	-	-	-
Bird 9a	-	-	-	-	-	-	0.71 (0.71-0.71)
Bird 9b	-	-	-	-	-	-	0.73 (0.73-0.73)

Tables S2. Comparison of *Campylobacter porA* fragment populations amongst faecal and caecal contents samples from individual broiler and breeder birds using the Bray-Curtis dissimilarity index. The calculations were performed on subsampled data to allow fair comparison between samples. A value of 0 indicates that two samples have the same *Campylobacter porA* fragment genotypes, and a value of 1 indicates that two samples do not share any *Campylobacter porA* fragment genotypes.

Broiler flocks

Subsampled to 1000 sequences

Flock A faeces

	Bird 1	Bird 2	Bird 3	Bird 4	Bird 5
Bird 1	0				
Bird 2	0.008	0			
Bird 3	0.006	0.005	0		
Bird 4	NT	NT	NT		
Bird 5	0.005	0.004	0.007	NT	0

Flock B faeces

	Bird 1	Bird 2	Bird 3	Bird 4	Bird 5
Bird 1	0				
Bird 2	0.37	0			
Bird 3	0.34	0.07	0		
Bird 4	0.34	0.53	0.47		
Bird 5	0.4	0.02	0.07	0.54	0

Flock C faeces

	Bird 1	Bird 2	Bird 3	Bird 4	Bird 5
Bird 1	0				
Bird 2	0.04	0			
Bird 3	0.15	0.17	0		
Bird 4	0.19	0.21	0.04	0	
Bird 5	0.15	0.17	0.003	0.04	0

Flock A caecal contents

	Bird 6	Bird 7	Bird 8	Bird 9	Bird 10
Bird 6	0				
Bird 7	0.77	0			
Bird 8	0.39	0.77	0		
Bird 9	0.2	0.59	0.39	0	
Bird 10	0.66	0.17	0.66	0.49	0

Flock B caecal contents

	Bird 6a	Bird 6b	Bird 7a	Bird 7b
Bird 6a	0			
Bird 6b	0.11	0		
Bird 7a	0.1	0.03	0	
Bird 7b	0.16	0.07	0.08	0

Flock D caecal contents

	Bird 6a	Bird 6b	Bird 7a	Bird 7b	Bird 8
Bird 6a	0				
Bird 6b	0.31	0			
Bird 7a	0.39	0.12	0		
Bird 7b	0.4	0.13	0.003	0	
Bird 8	0.37	0.21	0.003	<0.001	0

Breeder flocks

Faecal samples subsampled to 19,000 sequences, caecal samples subsampled to 45000 sequer

Flock E faeces

	Bird 1	Bird 2	Bird 3	Bird 4
Bird 1	0			
Bird 2	0.65	0		
Bird 3	0.58	0.62	0	
Bird 4	NT	NT	NT	0

Flock F faeces

	Bird 1	Bird 2	Bird 3	Bird 4	Bird 5
Bird 1	0				
Bird 2	0.39	0			
Bird 3	0.67	0.56	0		
Bird 4	0.55	0.45	0.48	0	
Bird 5	0.58	0.45	0.52	0.48	0

Flock G faeces

	Bird 1	Bird 2	Bird 3	Bird 4	Bird 5
Bird 1	0				
Bird 2	0.55	0			
Bird 3	0.53	0.31	0		
Bird 4	0.58	0.47	0.38	0	
Bird 5	0.8	0.45	0.58	0.67	0

Flock F caecal contents

	Bird 6	Bird 7	Bird 8	Bird 9	Bird 10
Bird 6	0				
Bird 7	0.58	0			
Bird 8	0.25	0.48	0		
Bird 9	0.53	0.61	0.55	0	
Bird 10	0.62	0.5	0.62	0.56	0

Flock G caecal contents

	Bird 6	Bird 7	Bird 8	Bird 9a	Bird 9b
Bird 6	0				
Bird 7	0.37	0			
Bird 8	0.36	0.37	0		
Bird 9a	0.3	0.42	0.47	0	
Bird 9b	0.46	0.59	0.52	0.44	0

>

Table S3: Flock by flock comparison of *Campylobacter porA* fragment genotype populations identified amongst faecal and caecal samples from broiler and breeder flocks using the Bray-Curtis dissimilarity index. The total number of sequences for each sample was subsampled to a size of 10,000 to allow fair comparison between samples. A value of 0 indicates that two samples have the same *Campylobacter porA* fragment genotypes, and a value of 1 indicates that two samples do not share any *Campylobacter porA* fragment genotypes.

		**Broiler flocks**						**Breeder flocks**				
		FlockA faec	FlockB faec	FlockC faec	FlockA caec	FlockB caec	FlockD caec	FlockE faec	FlockF faec	FlockG faec	FlockF caec	FlockG caec
**Broiler flocks**	FlockA _faec	0										
	FlockB _faec	0.99	0									
	FlockC _faec	0.99	0.95	0								
	FlockA _caec	0.35	0.67	0.98	0							
	FlockB _caec	1.00	0.19	1.00	0.65	0						
	FlockD _caec	0.99	0.94	0.03	0.98	0.99	0					
**Breeder flocks**	FlockE _faec	0.96	0.72	0.90	0.60	0.77	0.85	0				
	FlockF _faec	0.97	0.78	0.87	0.82	0.78	0.82	0.60	0			
	FlockG _faec	0.98	0.75	0.95	0.80	0.76	0.91	0.62	0.29	0		
	FlockF _caec	0.75	0.91	0.95	0.73	0.73	0.90	0.54	0.43	0.50	0	
	FlockG _caec	0.98	0.46	0.97	0.68	0.49	0.93	0.55	0.58	0.55	0.67	0

Table S4. **Comparison of *Campylobacter porA* fragment populations identified by parallel sequencing between (i) DNA extracted directly from an individual sample and (ii) from DNA extracted from bacterial growth from the same sample on mCCDA (‘plate sweep’), using the Bray-Curtis dissimilarity index.** The calculations were performed on data subsampled to 1,000 sequences, to allow fair comparison between samples. A value of 0 indicates that two samples have the same *Campylobacter porA* fragment genotypes, and a value of 1 indicates that two samples do not share any *Campylobacter porA* fragment genotypes.

	**Faecal samles**		**Caecal contents samples**			
	**Flock A**	**Flock B**	**Flock A**		**Flock B**	
Bird 1	0.01	0.29	Bir d 6	0.67	Bird 6a	0.26
Bird 2	0.01	0.26	Bird 7	0.21	Bird 6b	0.26
Bird 3	0.01	0.05	Bird 8	0.47	Bird 7a	0.04
Bird 4	NT	0.50	Bird 9	0.68	Bird 7b	0.21
Bird 5	0.01	0.02	Bird 10	0.24		

Figure S1. Rarefaction curves for *Campylobacter porA* fragment types identified from (A) faecal and caecal contents samples from four broiler flocks and three broiler breeder flocks; and for (B) broiler flocks and (C) broiler breeder flocks; examining the cumulative effect of identifying *Campylobacter porA* fragment types from between one and five individual samples per flock. Please note it was not possible for results from broiler samples, broiler breeder faecal samples and broiler breeder caecal contents samples to all be displayed using the same axis scales. Faecal samples were not tested from Flock D and caecal contents samples were not available to be tested for flocks C and E.

(A)

Broiler flocks

Broiler breeder flocks

Faecal samples | Caecal samples | Faecal samples | Caecal samples

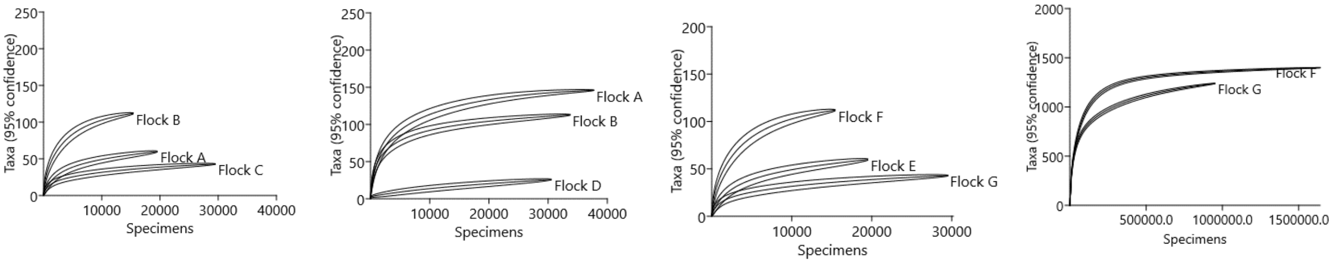

(B)

Broiler flock faecal samples

Flock A | Flock B | Flock C

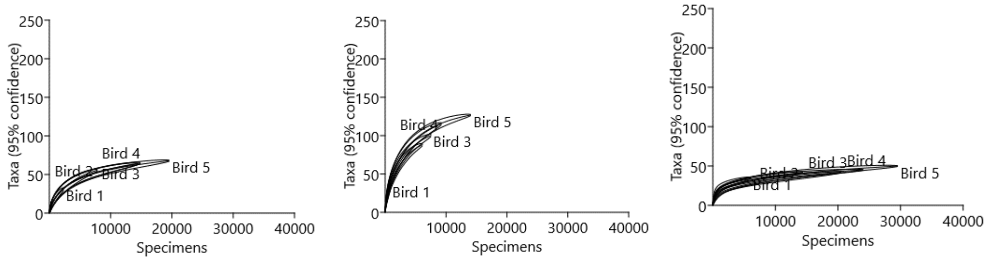

Broiler flock caecal contents samples

Flock A | Flock B | Flock D

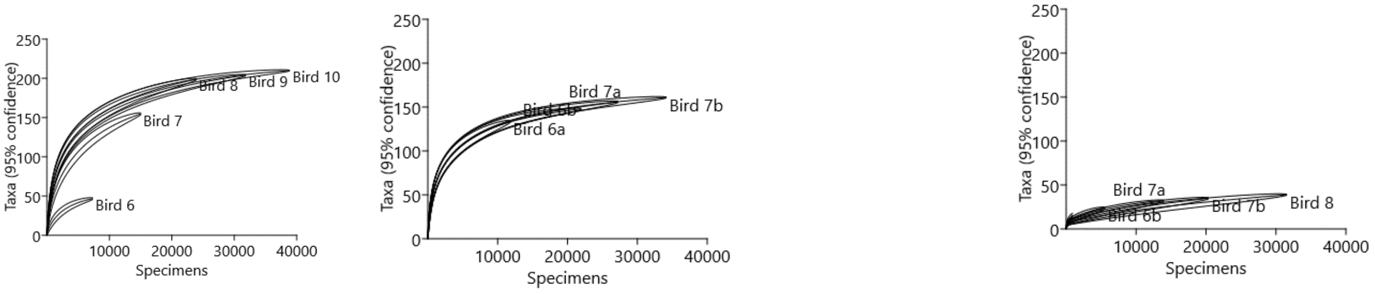

(C)

Breeder flock faecal samples

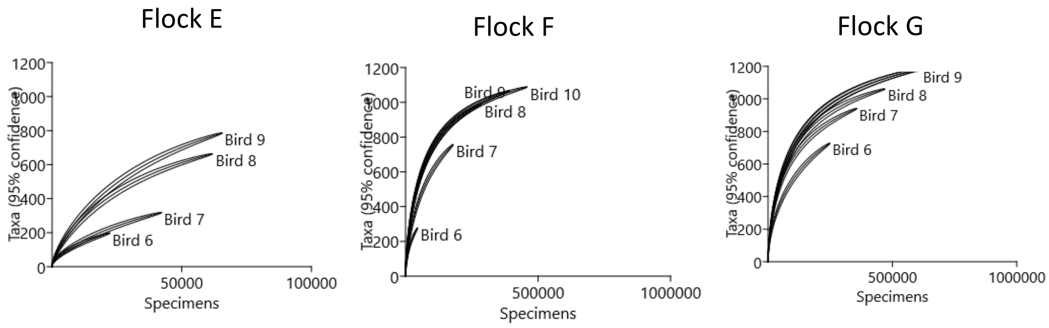

Breeder flock caecal contents samples

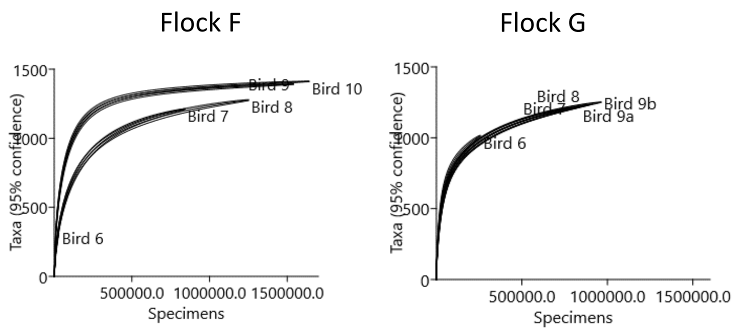

Supplement: Supplementary file 1 — Supplementary Dataset 1 [file 41598_2019_42207_MOESM1_ESM.pdf]
